# Whether AICAR in Pregnancy or Lactation Prevents Hypertension Programmed by High Saturated Fat Diet: A Pilot Study

**DOI:** 10.3390/nu12020448

**Published:** 2020-02-11

**Authors:** Wan-Long Tsai, Chien-Ning Hsu, You-Lin Tain

**Affiliations:** 1 Department of Pediatrics, Chiayi Chang Gung Memorial Hospital, Chiayi County, Puzi City 613, Taiwan; twldg007@gmail.com; 2 Department of Pharmacy, Kaohsiung Chang Gung Memorial Hospital, Kaohsiung 833, Taiwan; chien_ning_hsu@hotmail.com; 3 Department of Pediatrics, Kaohsiung Chang Gung Memorial Hospital and Chang Gung University College of Medicine, Kaohsiung 833, Taiwan

**Keywords:** AMP-activated protein kinase, asymmetric dimethylarginine, developmental origins of health and disease (DOHaD), high-fat, hypertension, nitric oxide, nutrient-sensing signals, oxidative stress

## Abstract

High consumption of saturated fats links to the development of hypertension. AMP-activated protein kinase (AMPK), a nutrient-sensing signal, is involved in the pathogenesis of hypertension. We examined whether early intervention with a direct AMPK activator 5-aminoimidazole-4-carboxamide riboside (AICAR) during pregnancy or lactation can protect adult male offspring against hypertension programmed by high saturated fat consumption via regulation of nutrient sensing signals, nitric oxide (NO) pathway, and oxidative stress. Pregnant Sprague–Dawley rats received regular chow or high saturated fat diet (HFD) throughout pregnancy and lactation. AICAR treatment was introduced by intraperitoneal injection at 50 mg/kg twice a day for 3 weeks throughout the pregnancy period (AICAR/P) or lactation period (AICAR/L). Male offspring (*n* = 7–8/group) were assigned to five groups: control, HFD, AICAR/P, HFD + AICAR/L, and HFD + AICAR/P. Male offspring were killed at 16 weeks of age. HFD caused hypertension and obesity in male adult offspring, which could be prevented by AICAR therapy used either during pregnancy or lactation. As a result, we demonstrated that HFD downregulated AMPK/SIRT1/PGC-1α pathway in offspring kidneys. In contrast, AICAR therapy in pregnancy and, to a greater extent, in lactation activated AMPK signaling pathway. The beneficial effects of AICAR therapy in pregnancy is related to restoration of NO pathway. While AICAR uses in pregnancy and lactation both diminished oxidative stress induced by HFD. Our results highlighted that pharmacological AMPK activation might be a promising strategy to prevent hypertension programmed by excessive consumption of high-fat food.

## 1. Introduction

Hypertension is a highly prevalent disease that can have a substantial impact on the global burden of cardiovascular disease in all world regions. The developmental origins of disease hypothesis (DOHaD) suggests that the early life environment plays a key role in the early origins of hypertension [1,2]. Blood pressure (BP) is regulated by a complex process that contains key contributions from the kidney. Developmental programming of renal structure and function, namely renal programming [3], increases the risk for developing hypertension in adult life. Conversely, reprogramming intervention aimed at reversing the programming processes, preceding the onset of hypertension, to prevent or delay the development of hypertension [4].

A high-fat diet is commonly used in animal models to induce obesity-related diseases, such as hypertension [5]. Maternal high-fat intake has been reported to induce an increase [6,7] or no change [7,8] on offspring’s BPs, mainly depending on the age, sex, and diverse fatty acids compositions [9]. Accumulative evidence indicates that diets containing high saturated fatty acids cause obesity/metabolic risk phenotypes, while high-fat diets based on poly-unsaturated fatty acids relates to beneficial effects [10]. Our previous study showed maternal and post-weaning high saturated fat (coconut oil-based) diets induced elevation of BP and kidney damage in male offspring at 24 weeks of age [7].

Dysregulated nutrient-sensing signals and impaired asymmetric dimethylarginine (ADMA, an endogenous inhibitor of nitric oxide synthase)-nitric oxide (NO) pathway are the proposed mechanisms underlying renal programming and programmed hypertension [11,12]. Fetal metabolism and development in response to maternal nutritional insults is mainly modulated by nutrient-sensing signaling pathways. In the kidney, several nutrient-sensing signals exist, including silent information regulator transcript (SIRT), cyclic adenosine monophosphate (AMP)-activated protein kinase (AMPK), peroxisome proliferator-activated receptors (PPARs), and PPARγ coactivator-1α (PGC-1α) [13]. Among them, AMPK is a serine/threonine protein kinase that serve as a central hub. AMPK is a heterotrimer composed of a catalytic α subunit in complex with two regulatory subunits, β and γ. All subunits exist in form of different isoforms (α1, α2, β1, β2, γ1, γ2, and γ3) [14]. AMPK is involved in BP control and renal physiology [15]. Additionally, AMPK has been shown to elicit antioxidant effects and regulate NO production [16].

HF diet was reported to reduce AMPK activity [17]. We previously observed that maternal metformin, an indirect AMPK activator, protects adult offspring against hypertension programmed by HFD [18]. Although a direct AMPK activator 5-aminoimidazole-4-carboxamide riboside (AICAR) has been reported to lower BP in adult spontaneously hypertensive rats (SHRs) [19], its reprogramming effect on programmed hypertension has not been examined yet. Thus, we hypothesized that maternal and post-weaning high saturated fat diets induced programmed hypertension via reducing AMPK and its related signals, whereas AICAR can prevent adult offspring against hypertension programmed by HFD.

## 2. Materials and Methods

### 2.1. Animal Models

The investigation conformed to the Institutional Animal Care and Use Committee of Kaohsiung Chang Gung Memorial Hospital (Permit number: 2018061302) that complies with the Guide for the Care and Use of Laboratory Animals of the National Institutes of Health. Virgin Sprague-Dawley (SD) rats (12–16 weeks old) were obtained from BioLASCO Taiwan Co., Ltd. (Taipei, Taiwan). Animals were maintained in an AAALAC-accredited animal facility, housed in a 12 h light/12 h dark cycle condition with a relative humidity of 55%. Rats were permitted ad libitum access to tap water and standard rat chow. Male SD rats were housed with individual females. Mating was confirmed by the examination of a vaginal plug.

**Figure 1:**
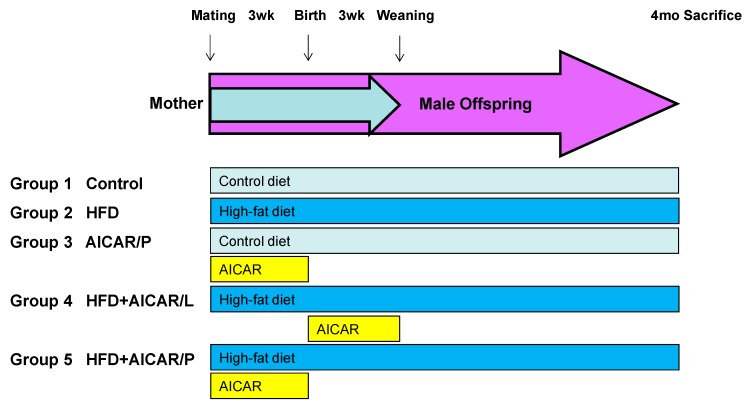
Animal protocol used in the present study.

Maternal rats received either a control diet with regular rat chow (Fwusow Taiwan Co., Ltd., Taichung, Taiwan; 52% carbohydrates, 23.5% protein, 4.5% fat, 10% ash, and 8% fiber) or high-fat diet (HFD; D12331, Research Diets, Inc., New Brunswick, NJ, USA; 58% fat (hydrogenated coconut oil) plus high sucrose (25% carbohydrate)) during pregnancy and lactation periods. In order to equal the received quantity of milk and maternal pup care, litters were standardized to eight pups per litter at birth. Because males are likely to have higher risk for developing hypertension younger than females [20], only male offspring were selected from each litter and used in subsequent experiments. Male offspring received either a control diet or HFD from post-weaning to 4 months of age. AICAR treatment was introduced by intraperitoneal injection at 50 mg/kg twice a day for 3 weeks throughout the pregnancy period (AICAR/P) or lactation period (AICAR/L) mixed in 0.9% sterile saline solution, and controls were treated with a 0.9% sterile saline solution vehicle. Another control group received AICAR treatment during the pregnancy period. The dose of AICAR used in pregnant rats was based on a previous study [21]. Male offspring were assigned to five groups (*n* = 7–8 for each group): control, HFD, AICAR/P, HFD + AICAR/L, and HFD + AICAR/P. Experimental protocol is illustrated in Figure 1. Only mother rats were given AICAR during pregnancy or lactation period. Male offspring was not treated with AICAR.

BP was measured in conscious and previously trained rats by using an indirect tail-cuff method (BP-2000, Visitech Systems, Inc., Apex, NC, USA) at 4, 8, 12, and 16 weeks of age [7]. The rats were acclimated to restraint and tail-cuff inflation for 1 week prior to the measurement, to ensure accuracy and reproducibility. BP measurements were taken between 13.00 and 17.00 each day on a blinded basis by the same experienced research assistant. Rats were placed on specimen platform, and their tails were passed through tail cuffs and secured in place with tape. Following a 10 min warm-up period, ten preliminary cycles of tail-cuff inflation were performed to allow the rats to adjust to the inflating cuff. For each rat, five cycles were recorded at each time point. Average of values from three stable measurements was taken. Male offspring were killed at 16 weeks of age. An intraperitoneal injection of ketamine (50 mg/kg) and xylazine (10 mg/kg) were used to assess anesthesia. Rats were then euthanized by an intraperitoneal overdose of pentobarbital. Kidneys and heparinized blood samples were collected at the end of the study.

### 2.2. High-Performance Liquid Chromatography (HPLC)

The plasma levels of several components of the NO pathway, including L-citrulline (the precursor of L-arginine), L-arginine (the substrate for NO synthase), ADMA, and SDMA (an isomer of ADMA), were measured using HPLC with the *o*-phtalaldehyde-3-mercaptoprionic acid derivatization reagent [7]. Concentrations of 1–100 mM L-citrulline, 1–100 mM L-arginine, 0.5–5 mM ADMA, and 0.5–5 mM SDMA were used as standards. The recovery rate was approximately 95%.

### 2.3. Quantitative Real-Time Polymerase Chain Reaction (PCR)

RNA was extracted from the kidney cortex according to previously described methods [7]. Several genes related to the nutrient sensing signaling pathway were analyzed in this study, including SIRT-1 (*Sirt1*), SIRT-4 (*Sirt4*), AMPK-α2 (*Prkaa2*), -β2 (*Prkab2*), and -γ2 (*Prkag2*), PPAR-α (*Ppara*), -β (*Pparb*), and -γ (*Pparg*), and PGC-1α (encoded by *Ppargc1a*). The 18S rRNA gene (*Rn18s*) was used as a reference gene. Primer sequences are provided in Table 1. RNA expression levels were normalized to 18S rRNA levels and calculated according to the ∆∆ comparative threshold (C_T_) method. Values were then calculated relative to control to generate a ΔΔC_T_ value. The fold-increase of the experimental sample relative to the control was calculated using the formula 2^−ΔΔCT^.

### 2.4. Western Blot

Samples were subjected to electrophoresis, Western blot, and antibodies incubation using the methods published previously [7]. Briefly, 200 μg of kidney cortex were loaded on a 6–10% polyacrylamide gel and separated by electrophoresis (200 volts, 90 min). Following transfer to a nitrocellulose membrane (GE Healthcare Bio-Sciences Corp., Piscataway, NJ, USA), the membranes were incubated with Ponceau S red (PonS) stain solution (Sigma-Aldrich, St. Louis, MO, USA) for 10 min on the rocker to verify equal loading. After blocking with phosphate-buffered saline–Tween (PBS-T) containing 5% dry milk, the membranes were incubated with primary antibody. The blots were incubated overnight at 4 °C with a 1:1000 dilution of anti-phosphorylated AMPKα (Thr172) antibody (1:1000, #2535, Cell Signaling, Danvers, MA, USA). Following five washes with 0.1% Tween-Tris-buffered saline (TBS-T), the membranes were incubated for 1 h with horseradish peroxidase-labeled secondary antibody diluted 1:1000 in TBS-T. Bands were visualized using SuperSignal West Pico reagent (Pierce; Rockford, IL, USA) and quantified by densitometry as integrated optical density (IOD). IOD was then normalized to total protein PonS staining. Protein abundance was expressed as IOD/PonS.

### 2.5. Immunohistochemistry Staining

Paraffin-embedded kidney tissue sectioned at 3-μm thickness was deparaffinized in xylene and rehydrated in a graded ethanol series to phosphate-buffered saline. 8-Hydroxydeoxyguanosine (8-OHdG) is a DNA oxidation product that was measured to assess DNA damage. Nutrient sensing signal AMPKα2 and PGC-1α were also analyzed by immunohistochemistry. Following blocking with immunoblock (BIOTnA Biotech., Kaohsiung, Taiwan), the sections were incubated for 2 hr at room temperature with an anti-8-OHdG antibody (1:100, JaICA, Shizuoka, Japan), an anti-AMPKα2 antibody (1:400, Proteintech, Rosemont, IL, USA), and an anti-PGC-1α antibody (1:200, Abcam, Cambridge, MA, USA). Immunoreactivity was revealed using the polymer-horseradish peroxidase (HRP) labelling kit (BIOTnA Biotech). For the substrate-chromogen reaction, 3,3′-diaminobenzidine (DAB) was used. Identical staining protocol omitting incubation with primary antibody was employed to prepare samples that were used as negative controls. Quantitative analysis of positive cells per microscopic field (X400) in the renal sections was performed as we described previously [7].

### 2.6. Statistical Analysis

Values given in figures and tables represent mean ± standard error of mean (SEM). Statistical significance was determined using one-way ANOVA with a post-hoc Tukey test for multiple comparisons. In all cases, a *p*-value less than 0.05 was considered statistically significant. All analyses were performed using the Statistical Package for the Social Sciences software (SPSS, Chicago, IL, USA).

## 3. Results

HFD and AICAR treatment had no effect on the survival of male pups. Consumption of HF diet caused a greater body weight (BW) compared to controls, which was prevented by AICAR treatment in lactation (Table 2). The kidney weights and the ratios of kidney weight-to-body weight were lower in the HFD, HFD + AICAR/L, and HFD/AICAR/P groups compared to controls. AICAR treatment in lactation did not affect the kidney weight and the ratio of kidney weight-to-body weight vs. the control. At 16 weeks of age, a significant elevation of systolic BP (~20 mmHg) was noted in the HFD group compared with the other four groups.

**Figure 2:**
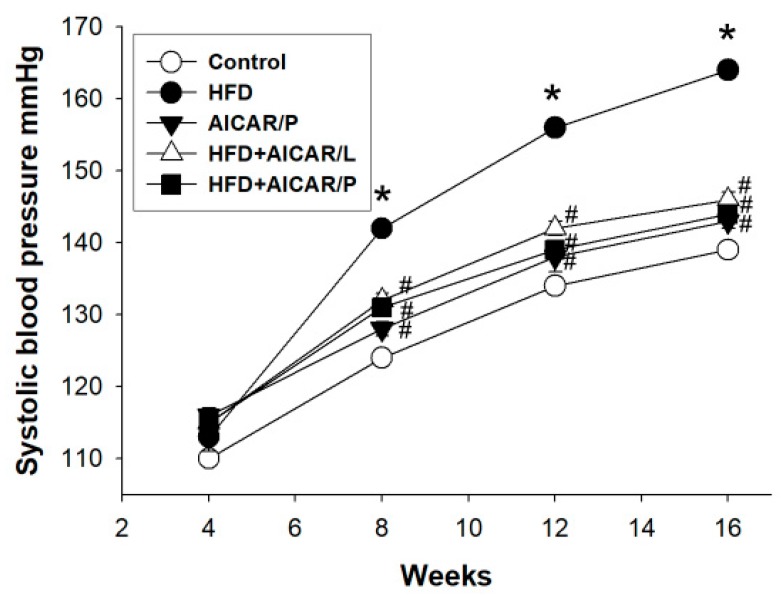
Effect of high-fat diet (HFD) and AICAR treatment in pregnancy (AICAR/P) or lactation (AICAR/L) on systolic blood pressure in male offspring. * *p* < 0.05 vs. control, # *p* < 0.05 vs. HFD.

As shown in Figure 2, systolic BP significantly increased in HFD group compared with that in control group from week 8 through 16. Compared to controls, AICAR treatment in pregnancy had no effect on systolic BP. While systolic BP was reduced by AICAR therapy in the HFD + AICAR/L and HFD + AICAR/P groups compared to that in the HDF group from 8 to 12 weeks of age. These data indicated that AICAR treatment either in pregnancy or lactation had similar protective effects on hypertension programmed by HFD.

**Figure 3:**
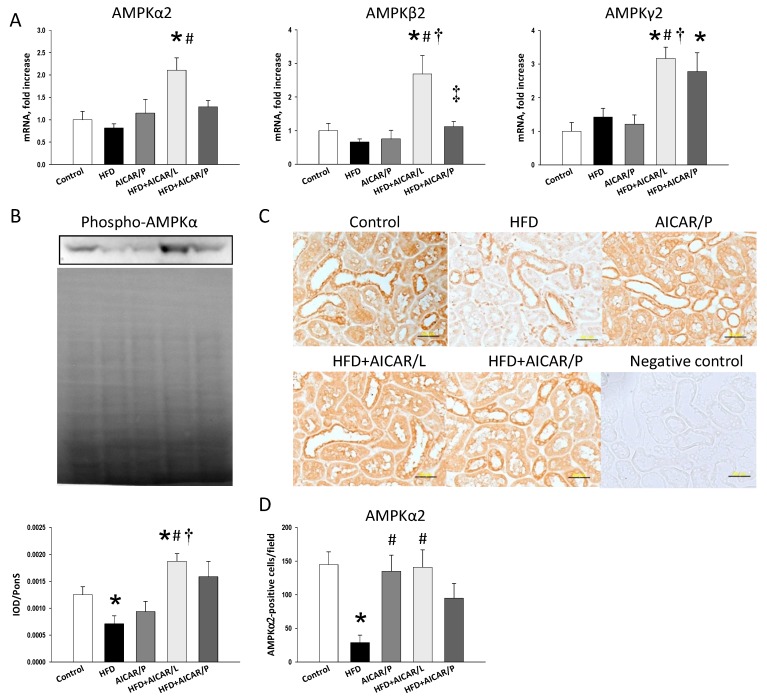
Effect of high-fat diet (HFD) and AICAR treatment in pregnancy (AICAR/P) or lactation (AICAR/L) on (**A**) mRNA expression of AMP-activated protein kinase (AMPK) α-, β-, and γ-subunits; (**B**) protein level of phosphorylated AMPKα (62 kDa) with represented blot and Ponceau S red (PonS) staining; (**C**) light microscopic findings of AMPKα2 immunostaining in the kidney cortex in 16-week-old male offspring; and (**D**) quantitative analysis of AMPKα2-positive cells per microscopic field (×400). Bar = 50 μm; * *p* < 0.05 vs. control, # *p* < 0.05 vs. HFD, † *p* < 0.05 vs. AICAR/P.

We first evaluated the mRNA and protein level of AMPK (Figure 3). As a result, we demonstrated that HFD or AICAR in pregnancy had neglectable effect on renal mRNA expression of AMPK-α2, -β2, and -γ2. However, AICAR treatment in lactation significantly increased AMPK-α2, -β2, and -γ2 mRNA expression compared to the control as well as HFD group (Figure 3A). Also, AICAR treatment in pregnancy caused a higher AMPK-γ2 mRNA expression in the HFD + AICAR/P group than that in the controls. Additionally, HFD reduced phosphorylated AMPKα protein abundance compared to the controls (Figure 3B). Consistent with the change in mRNA level, AICAR treatment in lactation significantly increased the renal protein level of phosphorylated AMPKα in offspring kidneys.

We next analyzed AMPKα2 (Figure 3C) using the immunohistochemical examination of renal sections. Immunostaining of AMPKα2 in the glomeruli and renal tubules indicated intense staining in the control (145 ± 19 positive cells), AICAR/P group (135 ± 24 positive cells), and HFD + AICAR/L group (141 ± 26 positive cells), an intermediate level of staining in the HFD + AICAR/P group (95 ± 22 positive cells), and little staining in the HFD group (29 ± 11 positive cells) (Figure 3C).

**Figure 4:**
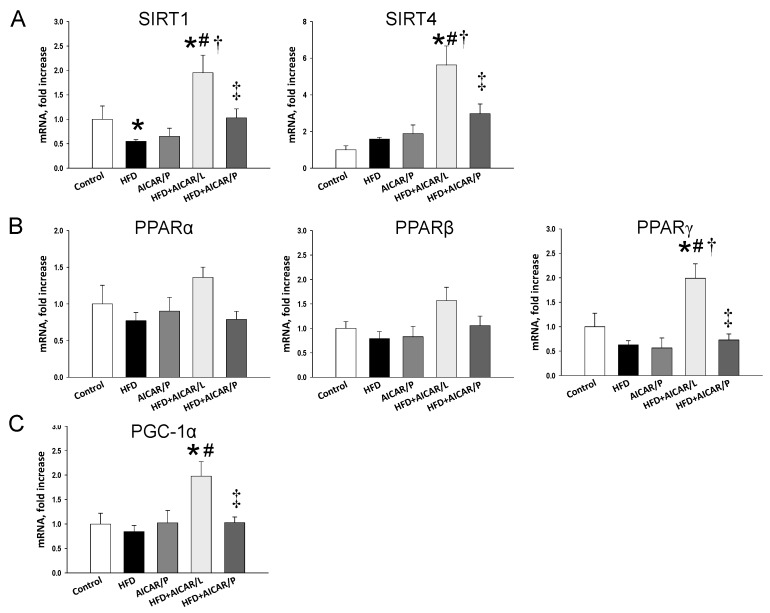
Effect of high-fat diet (HFD) and AICAR treatment in pregnancy (AICAR/P) or lactation (AICAR/L) on mRNA expression of (**A**) silent information regulator transcript 1 (SIRT1) and 4 (SIRT4); (**B**) peroxisome proliferator-activated receptor (PPAR) α-, β-, and γ-isoforms; and (**C**) PPARγ coactivator-1α (PGC-1α) in 16-week-old male offspring kidneys. * *p* < 0.05 vs. control, # *p* < 0.05 vs. HFD, † *p* < 0.05 vs. AICAR/P, ‡ *p* < 0.05 vs. HFD + AICAR/L.

**Figure 5:**
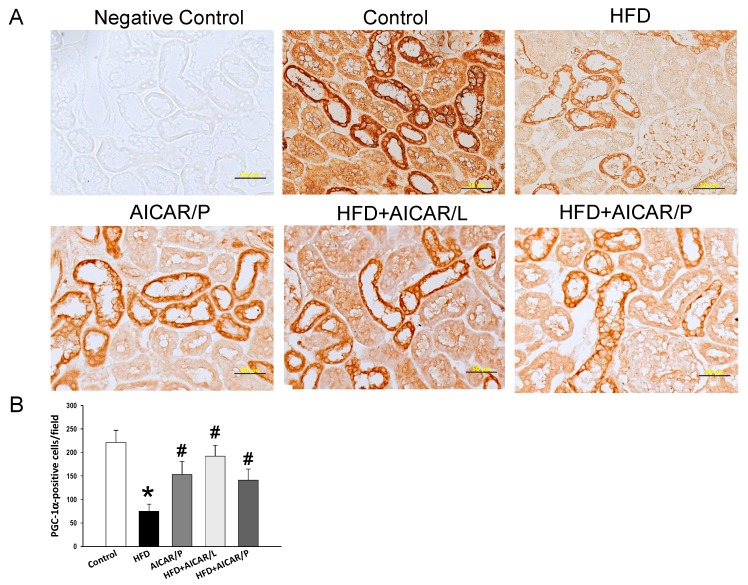
(**A**) Light microscopic findings of PPAR coactivator-1α (PGC-1α) immunostaining in the kidney cortex in 16-week-old male offspring. Bar = 50 μm; (**B**) quantitative analysis of PGC1α-positive cells per microscopic field (400×); * *p* < 0.05 vs. control, # *p* < 0.05 vs. HFD.

As AMPK is a key nutrient-sensing signal, we next analyzed the genes involved in the nutrient sensing pathway. As shown in Figure 4, HFD reduced renal mRNA expression of *Sirt1*. AICAR therapy in pregnancy significantly increased *Sirt1*, *Sirt4*, *Pparg* (encoding for PPARγ), and *Ppargc1a* (encoding for PGC-1α) in HFD + AICAR/P group compared to the other four groups. While AICAR treatment in pregnancy, unlike in lactation, had a negligible effect on nutrient-sensing signals. Also, we analyzed PGC-1α in the offspring kidneys by immunohistochemistry (Figure 5). Similar to phosphorylated AMPKα, AICAR treatment in lactation attenuated the reduction of PGC-1α expression caused by HFD (control: 221 ± 26 positive cells; HFD group: 75 ± 15 positive cells; HFD + AICAR/L group: 192 ± 23 positive cells). AICAR treatment in pregnancy also increased the immunostaining of PGC-1α (141 ± 24 positive cells) in the HFD + AIRCA/P group compared to that in the HFD group (Figure 5). Taken together, these findings indicated that HFD downregulated AMPK/SIRT1/PGC-1α pathway, which can be restored by AICAR treatment in lactation and to a lesser extent in pregnancy.

Because the interplay between ADMA-NO pathway and oxidative stress contributes to the pathogenesis of programmed hypertension [11,12], we further investigated whether AICAR treatment can mediate ADMA-NO pathway to prevent hypertension programmed by HFD (Table 3). Our results showed that plasma L-citrulline level (the precursor of L-arginine) did not differ among the five groups. HFD caused the decreases of plasma L-arginine level (the substrate for NO synthase) and L-arginine-to-ADMA ratio, an index of NO bioavailability [22]. AICAR therapy in pregnancy caused higher plasma L-arginine and ADMA levels in the AICAR/P groups than those in controls. AICAR therapy in lactation restored the increases of plasma SDMA levels induced by HFD. Additionally, AICAR therapy in pregnancy results in a higher plasma L-arginine level but a lower SDMA level in the HFD + AICAR/P groups than those in the HFD group.

**Figure 6:**
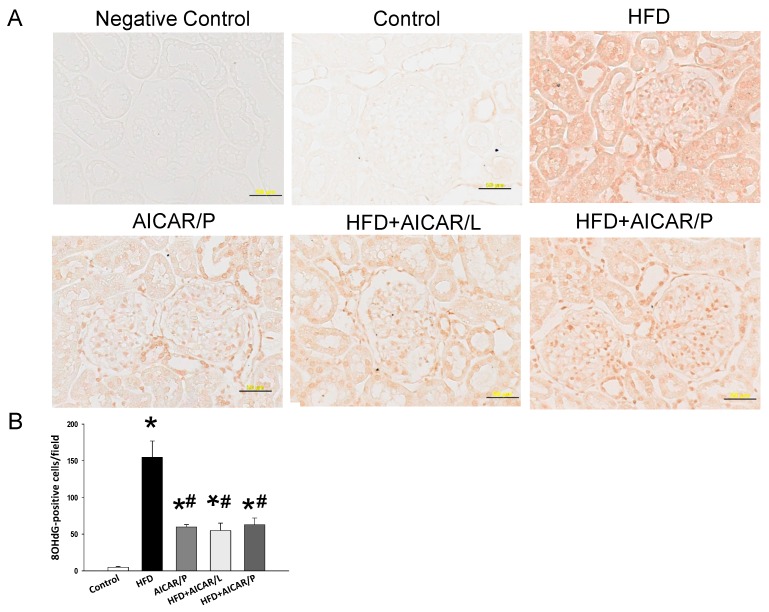
(**A**) Light microscopic findings of 8-hydroxydeoxyguanosine (8-OHdG) immunostaining in the kidney cortex in 16-week-old male offspring. Bar = 50 μm; (**B**) quantitative analysis of 8-OHdG-positive cells per microscopic field (×400); * *p* < 0.05 vs. control, # *p* < 0.05 vs. HFD.

We further assessed oxidative stress by immunostaining of 8-hydroxydeoxyguanosine (8-OHdG), a metabolite of oxidative damage to leukocyte DNA. Cytoplasmic and nuclear staining was present with little staining in the control group (5 ± 1 positive cells), a similar intermediate intensity in the AICAR/P (60 ± 3 positive cells), HFD + AICAR/L (55 ± 10 positive cells), and HFD + AICAR/P group (63 ± 9 positive cells), and intense staining in the HFD (155 ± 22 positive cells) (Figure 6). Additionally, HFD + AICAR/P group had a higher L-arginine level, and a lower ADMA level and L-arginine-to-ADMA ratio than those in the HFD + AICAR/L group. Taken together, our findings implied that AICAR therapy in pregnancy protects hypertension programmed by HFD is related to restoration of ADMA-NO pathway. Unlike its use in pregnancy, AICAR treatment in lactation had neglectable effect on ADMA-NO pathway.

## 4. Discussion

Our study describes, for the first time, direct AMPK activation using its activator AICAR protects male offspring against hypertension programmed by maternal plus post-weaning high saturated fat intake and puts special emphasis on the analysis of nutrient-sensing signals and oxidative stress. The major findings of this study were: (1) maternal plus post-weaning HFD resulted in hypertension and obesity in male adult offspring, which could be prevented by AICAR therapy used either during gestation or lactation; (2) HFD induced programmed hypertension is related to downregulation of AMPK/SIRT1/PGC-1α pathway; (3) therapeutic use of AICAR in lactation, to a greater extent than in pregnancy, activated AMPK signaling pathway; (4) the anti-hypertensive effect of AICAR therapy used in pregnancy and/or lactation is, at least in part, due to restoration of ADMA-NO pathway; and (5) both AICAR uses in pregnancy and lactation attenuated oxidative stress programmed by HFD in offspring kidneys.

Unlike other direct AMPK activators show isoform-specific activations, AICAR is a potent pan-activators for all 12 heterotrimetric AMPK complexes [23]. In line with previous studies using indirect AMPK activators [18,24,25,26], this is the first report of AICAR therapy activating AMPK signaling pathway to prevent hypertension of developmental origins [26]. We observed that the anti-hypertensive effect of AICAR either used during pregnancy or lactation was starting in week 8 and over time. Results from the present study support the argument that the reduction of BP is mainly due to reprogramming effect of AICAR instead of its acute hypotensive effect. Although AICAR has been shown good safety in a clinical trial to treat cancer patients [27], at high concentration it is toxic for mammalian cells [28]. As a result, we demonstrated that AICAR therapy in pregnancy or lactation showed similar offspring’s BP. Of note is that AICAR use in lactation had a greater effect on activating AMPK signaling pathway than in pregnancy. As AICAR can induce AMPK activity in placenta [21], its transfer and metabolism by the placenta might explain the discrepancy. However, additional studies are required to elucidate the ideal dose and timing for AICAR use in programmed hypertension.

The results of this study showed that one positive effect of AICAR is relevant to activation of the AMPK/SIRT1/PGC-1α pathway. Early-life environmental insults can program AMPK and other nutrient-sensing signals to regulate PPARs and their target genes, hence provoking programmed hypertension [29]. Since that systemic BP was higher in AMPKα2 knockout mice than in wildtype mice [30], and that our previous studies showed AMPKα2 protein is related to programmed hypertension [25,31], we mainly focused on determining AMPKα2 protein level in the current study. Our previous study reported that resveratrol, a known natural activator of AMPK, prevents hypertension programmed by HFD associated with increased protein levels of SIRT1 and AMPKα2 [25]. Additionally, we recently found that resveratrol therapy activated AMPK/SIRT1/PGC-1α pathway and protected offspring against hypertension and oxidative stress in another hypertension model of programming [31].

Given that resveratrol has multifaceted biological functions, its reprogramming effects might not directly relate to AMPK activation. Results of the current study go beyond previous reports, showing that therapeutic use of direct AMPK activator AICAR in lactation activated AMPK/SIRT1/PGC-1α pathway and prevented the development of hypertension concurrently. Although AICAR use in pregnancy had a minor effect of mRNA expression of several nutrient-sensing signals and protein level of AMPKα2 compared to its use in lactation, both treatments displayed similar impacts on protein levels of PGC-1α. Future research should certainly further test whether other isoform-specific AMPK activators can serve as potential intervention to reverse the programming processes to prevent the developmental programming of hypertension and examine them in different kinds of programmed hypertension models.

Another beneficial effect of AICAR is via restoration of NO system. Our earlier report showed that metformin, an indirect AMPK activator, prevents the development of hypertension in spontaneously hypertensive rats via reducing ADMA and increasing NO production [32]. In the current study, AICAR therapy in pregnancy prevents hypertension that is associated with increased plasma levels of L-arginine and L-arginine-to-ADMA ratios, but decreased plasma levels of ADMA and SDMA. Since L-arginine-to-ADMA ratios represent NO bioavailability [22], both ADMA and SDMA are inhibitors of NO synthase [33], signals formed by NO pathway are supposed to cause vasodilatation. Therefore, our data are in accordance with the previous findings reported that AICAR ameliorates portal hypertension via preserving NO pathway [34]. We conducted the AICRA/P group to evaluate the programming effect of AICAR in control offspring. Although AICAR use in pregnancy had neglectable effects on BP and nutrient-sensing signal pathway in controls, our results showed higher plasma L-arginine and ADMA levels and greater intensity of 8-OHdG staining in the AICAR/P group compared to controls. Thus, whether AICAR use in pregnancy might induce other programming effects in control offspring deserves further clarification.

In this work, another preventive effect of AICAR against HFD-induced programmed hypertension might be, at least in part, due to reduction of oxidative stress. Oxidative stress is a well-known mechanism involved in the developmental programming of hypertension [12]. AMPK activation has been shown to suppress pro-oxidant enzymes and upregulate anti-oxidant enzymes, to reduce oxidative stress [35,36]. Our data demonstrated the presence of 8-OHdG staining, an oxidative stress damage marker, in the offspring exposed to HFD, which was attenuated by AICAR therapy. A similar pattern of results was obtained from AICAR therapy either used during pregnancy or lactation period. These findings support the notion that AMPK activation by AICAR in early life could prevent HFD-induced oxidative stress in adult offspring.

Our study has some limitations that are worth noting. Although current research is geared primarily to finding the beneficial effect of AICAR in the kidneys, its protective effect may come from other organs that regulate BP, such as the brain, the heart, and the vasculature. Although AMPK activation has been initially recognized as a dominant effect of AICAR, this compound also triggers AMPK-independent effects [37]. Further studies should investigate organ- and isoform-specific effects of AICAR and other AMPK activators to clarify their relationships with programmed hypertension in different models of programmed hypertension. Second, we did not examine the different doses of AICAR, regardless of we did test AICAR in two therapeutic durations. Although therapeutic uses of AICAR in pregnancy or lactation exert similar BP-lowering effects, they had differential impacts on NO system and nutrient-sensing signals. This is an interesting topic for future work. We did not conduct a control group that received AICAR treatment during lactation because we have conducted the AICAR/P group to evaluate programming effects of AICAR on normal control offspring. Nevertheless, the programming effects of AICAR treatment during pregnancy or lactation might be different and that deserve further elucidation. Lastly, we did not evaluate sex difference in response to AICAR, as only male offspring were recruited in this study. The reason for this is due to the nature of programmed hypertension, hypertension occurred at an early age in males than females [20] and we measured BP in young adulthood.

## 5. Conclusions

In summary, the current findings cast a new light on therapeutic uses of AICAR in pregnancy or lactation to prevent hypertension programmed by HFD. Our results lend additional support to the notion that pharmacological AMPK activation can be a possible reprogramming strategy to improve the alarming scenario of hypertension and its related disorders. The ultimate challenge will be successful translation of the promising preclinical findings from animal studies into practical clinical applications.

## Figures and Tables

**Table 1:** Quantitative real-time polymerase chain reaction primers sequences.

Gene		Reverse
*Sirt1*	5 tggagcaggttgcaggaatcca 3	5 tggcttcatgatggcaagtggc 3
*Sirt4*	5 ccctttggaccatgaaaaga 3	5 cggatgaaatcaatgtgctg 3
*Prkaa2*	5 agctcgcagtggcttatcat 3	5 ggggctgtctgctatgagag 3
*Prkab2*	5 cagggccttatggtcaagaa 3	5 cagcgcatagagatggttca 3
*Prkag2*	5 gtgtgggagaagctctgagg 3	5 agaccacacccagaagatgc 3
*Ppara*	5 agaagttgcaggaggggatt 3	5 ttcttgatgacctgcacgag 3
*Pparrb*	5 gatcagcgtgcatgtgttct 3	5 cagcagtccgtctttgttga 3
*Pparg*	5 ctttatggagcctaagtttgagt 3	5 gttgtcttggatgtcctcg 3
*Ppargc1a*	5 cccattgagggctgtgatct 3	5 tcagtgaaatgccggagtca 3
*Rn18s*	5 gccgcggtaattccagctcca 3	5 cccgcccgctcccaagatc 3

**Table 2:** Measures of body weight, kidney weight, and blood pressure in 16-week-old male offspring exposed to high-fat diet (HFD) and 5-aminoimidazole-4-carboxamide riboside (AICAR) in pregnancy or lactation.

Groups	Control	HFD	AICAR/P	HFD + AICAR/L	HFD + AICAR/P
Number	7	8	8	8	8
BW (g)	610 ± 12	793 ± 17 ^a^	606 ± 19	588 ± 26 ^b^	644 ± 22
Left kidney weight (g)	2.36 ± 0.06	1.7 ± 0.06 ^a^	2.02 ± 0.04	1.77 ± 0.07 ^a^	1.5 ± 0.08 ^a,c^
Left kidney weight/100 g BW	0.39 ± 0.01	0.31 ± 0.01 ^a^	0.36 ± 0.01	0.31 ± 0.02 ^a^	0.29 ± 0.01 ^a,c^
Systolic blood pressure (mm Hg)	139 ± 1	164 ± 1 ^a^	143 ± 1	146 ± 1 ^b^	144 ± 1 ^c^

HFD, high-fat diet; AICAR/P, AICAR treatment during pregnancy; HFD + AICAR/L, high-fat diet plus AICAR treatment during lactation; HFD + AICAR/P, high-fat diet plus AICAR treatment during pregnancy. BW, body weight; *n* = 7–8/group; ^a^ *p* < 0.05 vs. control; ^b^ *p* < 0.05 HFD vs. HFD + AICAR/L; ^c^ *p* < 0.05 HFD vs. HFD + AICAR/P.

**Table 3:** Plasma l-citrulline, l-arginine, ADMA, and SDMA levels in 16-week-old male offspring exposed to high-fat diet (HFD) and AICAR in pregnancy or lactation.

Groups	Control	HFD	AICAR/P	HFD + AICAR/L	HFD + AICAR/P
L-citrulline	59.7 ± 4.5	53.7 ± 3.4	55.1 ± 4.6	57.1 ± 3.1	62.7 ± 3
L-arginine	141.4 ± 6.2	104.6 ± 4.4 ^a^	171.2 ± 10.6 ^a^	115.5 ± 3.3 ^a^	140.2 ± 7.2 ^c^
ADMA	1.35 ± 0.12	1.42 ± 0.09	1.73 ± 0.11 ^a^	1.53 ± 0.06	1.19 ± 0.07
SDMA	0.57 ± 0.11	0.7 ± 0.03	0.7 ± 0.06	0.52 ± 0.02 ^b^	0.49 ± 0.05 ^c^
L-arginine-to-ADMA ratio	110 ± 12	75 ± 3 ^a^	99 ± 3	76 ± 3 ^a^	118 ± 4 ^c^

ADMA, asymmetric dimethylarginine; SDMA, symmetric dimethylarginine; HFD, high-fat diet; AICAR/P, AICAR treatment during pregnancy; HFD + AICAR/L, high-fat diet plus AICAR treatment during lactation; HFD + AICAR/P, high-fat diet plus AICAR treatment during pregnancy; *n* = 7/group; ^a^ *p* < 0.05 vs. control; ^b^ *p* < 0.05 HFD vs. HFD + AICAR/L; ^c^ *p* < 0.05 HFD vs. HFD + AICAR/P.
